# Blood neurofilament light chain as a biomarker for monitoring and predicting paclitaxel-induced peripheral neuropathy in patients with gynecological cancers

**DOI:** 10.3389/fonc.2022.942960

**Published:** 2022-08-17

**Authors:** Su-Hyun Kim, Ki Hoon Kim, Jae-Won Hyun, Ji Hyun Kim, Sang-Soo Seo, Ho Jin Kim, Sang-Yoon Park, Myong Cheol Lim

**Affiliations:** ^1^Department of Neurology, Research Institute and Hospital of National Cancer Center, Goyang, South Korea; ^2^Center for Gynecologic Cancer, National Cancer Center, Goyang, South Korea; ^3^Center for Clinical Trial, Hospital, National Cancer Center, Goyang, South Korea; ^4^Department of Cancer Control and Population Health, National Cancer Center Graduate School of Cancer Science and Policy, Goyang, South Korea; ^5^Rare and Pediatric Cancer Branch and Immuno-oncology Branch, Division of Rare and Refractory Cancer, Research Institute, National Cancer Center, Goyang, South Korea; ^6^Department of Cancer Control and Policy, National Cancer Center, Goyang, South Korea

**Keywords:** paclitaxel, neuropathy, neurofilament light (NfL), gynecological cancer, chemotherapy induced peripheral neuropathy

## Abstract

**Objective:**

We aimed to evaluate the potential of serum neurofilament light chain (sNfL) and serum brain-derived neurotrophic factor (sBDNF) as reliable biomarkers for paclitaxel-induced peripheral neuropathy (PIPN).

**Methods:**

Forty-eight patients with gynecologic cancer scheduled to undergo six cycles of paclitaxel-based chemotherapy at the National Cancer Center of Korea between September 2020 and January 2022 were prospectively assessed during and after chemotherapy.

**Results:**

At the end of the chemotherapy, 12 (25%) patients were classified as having grade 3 PIPN according to the National Cancer Institute-Common Toxicity Criteria. The sNfL levels increased during paclitaxel treatment in all patients. After two, four, and six cycles, patients with grade 3 PIPN exhibited higher mean sNfL levels than those in the 0–2 grade range (p = 0.004, p = 001, and p < 0.001, respectively). For sNfL levels ≥ 124 pg/mL, after two cycles of chemotherapy, the sensitivity and specificity for predicting grade 3 PIPN at the end of treatment were 80% and 79%, respectively. Over the course of paclitaxel-based treatment, sBDNF levels continued to decrease regardless of the severity of PIPN. At the end of treatment and six months after chemotherapy, patients with grade 3 PIPN had lower sBDNF levels than those within the 0–2 grade range (p =0.037 and 0.02, respectively), and the patients in the latter group had better clinical symptoms six months after the end of treatment.

**Conclusions:**

The sNfL levels during paclitaxel-based chemotherapy reflect ongoing neuroaxonal injury and serve as reliable biomarkers of PIPN severity. The sNfL levels during early treatment with paclitaxel might be prognostic indicators for PIPN progression. Low sBDNF levels 6 months after chemotherapy might adversely affect PIPN recovery.

## Introduction

Paclitaxel is used to treat solid tumors, including breast, gastrointestinal, genitourinary/gynecological, and lung cancers. It exerts its antitumor effect by enhancing microtubule assembly and stabilizing microtubules (1). Microtubules are essential for the development and maintenance of neurons; thus, paclitaxel causes peripheral neuropathy in up to 97% of patients who receive treatment, affecting their quality of life (2). The clinical presentation of paclitaxel-induced peripheral neuropathy (PIPN) includes length-dependent sensory axonal neuropathy with symmetric dysesthesia, pain, and sensory loss in a stocking-glove pattern. Motor and autonomic symptoms may also occur but are less common (2). The current methods for diagnosing and grading PIPN are not optimal (3). Physician-based instruments, such as the National Cancer Institute-Common Toxicity Criteria (NCI-CTC), show low interrater reliability and poor sensitivity to change (3–5). The total neuropathy score, which includes clinician-rated items, as well as a neurophysiological examination (nerve conduction study [NCS] and quantitative sensory testing), shows better interrater reliability, but these tests are not easily accessible for the evaluation of cancer patients in daily clinical practice, and the severity of symptoms is not always correlated with the NCS findings (6, 7). The lack of a sensitive, reliable, and easily accessible biomarker to measure peripheral nerve injury is a major obstacle for the objective evaluation of PIPN.

Neurofilament light chain (NfL) is a cytoskeleton protein expressed in large-caliber myelinated axons and is a potential blood biomarker for axonal degeneration in various neurological diseases, such as dementia, multiple sclerosis, and acquired peripheral neuropathies (8, 9). In a previous study, we showed that serum NfL (sNfL) is a useful biomarker for axonal injury in colon cancer patients with oxaliplatin-induced peripheral neuropathy (OIPN) (7). The sNfL levels increased during oxaliplatin treatment, and patients with severe OIPN showed significantly higher mean sNfL levels than patients with mild to moderate OIPN (7). In this study, we extended our evaluations to paclitaxel-treated gynecological cancer patients to demonstrate a correlation between sNfL levels and the severity of axonal damage in PIPN. In addition, brain-derived neurotrophic factor (BDNF) promotes neuronal survival, growth, and repair (10). BDNF may be involved in the development of chemotherapy-induced peripheral neuropathy (CIPN), and its blood concentration is inversely correlated with the severity of bortezomib-induced neuropathy (11, 12). In this study, we investigated the changes in serum BDNF in addition to sNfL levels during paclitaxel treatment and their association with the risk of PIPN.

## Materials and methods

### Patients

We prospectively studied patients with pathologically verified stage II–IV ovarian cancer or endometrial cancer (papillary serous or clear cell carcinoma) who were scheduled to receive paclitaxel-based chemotherapy after surgery between October 2020 and January 2022 at the National Cancer Center in South Korea. The eligibility criteria included normal hematological, renal, and liver function. The patients received six courses of paclitaxel (175 mg/m^2^) intravenously (IV) over 3 hours, carboplatin (area under the curve [AUC] 5 mg/mL/min) IV over 1 hour and/or bevacizumab (7.5 mg/kg) every 3 weeks. The exclusion criteria included known peripheral neuropathy, alcohol abuse, degenerative neurological disorders, and a history of brain injury within 1 year. Seven patients previously exposed to neurotoxic chemotherapeutic agents and two with well-controlled diabetes mellitus without neuropathic symptoms were enrolled. Among the 65 patients who were initially enrolled in this study, 17 were excluded for reasons of discontinuation of treatment (due to disease progression or patient decision, n = 7), consent withdrawal (n = 5), transfer to another hospital (n = 4), and death during treatment (n = 1). Serum samples were also collected from 68 healthy controls (HCs) (68 samples) between 40 and 70 years of age. The data used in the current study were collected between October 2020 and January 2022. The institutional Review Board of the National Cancer Center approved this study (NCC 2020-0202), and written informed consent was obtained from all patients.

### Evaluation of peripheral neuropathy

All patients underwent clinical examinations and NCS before chemotherapy treatment, after receiving six cycles of chemotherapy (end of treatment), and 6 months after the end of treatment. PIPN was defined as persistent, symmetrical distal paresthesia accompanied by pain and numbness (13). PIPN severity was categorized as grade 1, 2, or 3 by a neurologist (S.H.K), using version 3.0 of NCI-CTC (14). Patient-reported outcomes were also used to quantify the severity of PIPN using the European Organisation for Research and Treatment of Cancer Quality of Life-Chemotherapy-Induced Peripheral Neuropathy 20 module (EORTC QLQ-CIPN20) at baseline, after two, four, and six cycles, and 6 months after the end of treatment. The NCS was performed following a standard technique, as previously described (15). Amplitude of sensory nerve action potential (a-SAP) and sensory nerve conduction velocity (SCV) were recorded in the ulnar and sural nerves, while the compound muscle action potential (CMAP) and motor nerve conduction velocity (MCV) were recorded in the ulnar and tibial nerves.

### Measurement of sNfL and sBDNF concentrations

Blood samples from patients were serially collected before surgery (pre-op), 2–3 weeks after surgery (pre-chemotherapy), after two, four, and six cycles, and 6 months after the end of treatment. The collected samples were immediately separated by centrifugation and frozen at -80°C until analysis. Concentrations of sNfL and sBDNF were measured using the NF-Light™ advantage kit and BDNF discovery kit, respectively; both are operated on an HD-1 single molecule array platform (SIMOA; Quanterix, Lexington, MA, USA). All measurement procedures were performed according to the manufacturer’s instructions. The intra-assay and inter-assay coefficients of variation were < 10%.

### Statistical analysis

The Kolmogorov–Smirnov test was used to assess whether a set of variables exhibited normal distribution. Comparison of characteristics between the different PIPN severity groups was performed using the t-test or the Mann–Whitney test for continuous variables, or the Chi-squared test or Fisher’s exact test for categorical variables. The results are expressed as mean plus standard deviation (SD)/error or median plus interquartile range (IQR). Wilcoxon’s signed-rank test for paired observations was used to compare differences between the clinical and NCS data and serum protein levels from serial assessments. Spearman’s correlation analysis was used to investigate correlations between serum protein levels and age. Receiver operating characteristics (ROC) analyses were used to determine the optimal sNfL cut-off levels for the identification of grade 3 PIPN. Comparisons of sNfL levels and NCS data were conducted using analysis of covariance, with age as a covariate, and Bonferroni tests were used for *post-hoc* comparisons. Correlations between sNfL levels and clinical data were analyzed using partial correlation while adjusting for age. SAS software (version 9.3; SAS Institute, Cary, NC, USA) was used for all statistical analyses. Two-sided p values < 0.05 were considered statistically significant.

## Results

**Figure 1:**
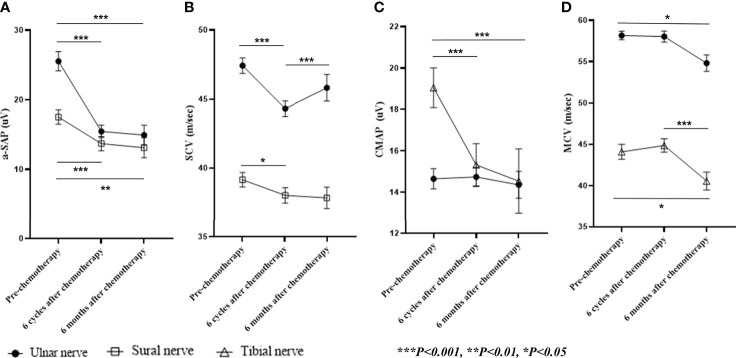
Nerve conduction studies. Shown are **(A)** a-SAP, **(B)** SCV in the ulnar and sural nerves, **(C)** CMAP, **(D)** MCV in the ulnar and tibial nerves during and six months after paclitaxel treatment. Data are presented as mean ± standard error. a-SAP, amplitude of the sensory nerve action potential; SCV, sensory nerve conduction velocity; CMAP, compound muscle action potential; MCV, motor nerve conduction velocity.

Forty-eight women [median (IQR) age: 54 (45–63) years] with ovarian or endometrial cancer were included in the study. The median (IQR) body surface area and cumulative paclitaxel doses were 1.57 (1.44–1.64) mg/m^2^ and 1040 (905–1056) mg/m^2^, respectively. The paclitaxel dose was reduced in six (13%) patients for causes unrelated to PIPN. Seven patients had had prior exposure to taxanes or platinum-based combination chemotherapy 2–7 years before the study. They had exhibited no symptoms or mild neuropathic symptoms during the previous chemotherapy round but had fully recovered and were not experiencing any discomfort at the time of enrollment in this study. At the end of chemotherapy, 96% patients presented PIPN, classified as grade 1 (31%, n = 15), 2 (40%, n = 19), and 3 (25%, n = 12). The EORTC QLQ-CIPN20 scores increased with the treatment cycles (**Table 1**), peaking after the fourth cycle and then remained the same until the end of therapy. Nineteen (40%) patients received neuropathic pain-modulating agents, including gabapentin, pregabalin, duloxetine, or tapentadol, after 2–4 cycles of chemotherapy. Six months after the end of treatment, the mean EORTC QLQ-CIPN20 scores decreased compared to those recorded at the end of chemotherapy (mean 23.31 ± 5.8 vs. mean 29.43 ± 9.79). However, these scores were still higher than the pre-chemotherapy scores (mean 19.85 ± 1.44); 42% of the patients had grade 1 PIPN, 25% had grade 2, and 8% had grade 3 at six months after the end of treatment. After six cycles of treatment, there was a significant decrease in the mean a-SAP and SCV of the ulnar and sural nerves (**Figures 1A, B**) and mean CMAP of the tibial nerve (**Figure 1C**), which indicates axon loss in sensory and motor nerves. Six months after the end of treatment, the mean a-SAP of the ulnar and sural nerves and the mean CMAP of the tibial nerve were still lower than those recorded at baseline. The mean MCV of the ulnar and tibial nerves did not change at the end of chemotherapy but decreased 6 months after chemotherapy (**Figure 1D**).

**Table 1:** Comparison of characteristics between grade 3 vs. grade 0–2 PIPN patients at the end of chemotherapy.

	Patients with grade 0–2 PIPN (n = 36)	Patients with grade 3 PIPN (n = 12)	p-value
**Age (years), mean ± SD**	51.8 ± 10.7	61.8 ± 3.9	**0.005**
**Type of gynecological cancer**			0.659
** Ovarian cancer, n (%)**	29 (81%)	11 (92%)	
** Endometrial cancer, n (%)**	7 (19%)	1 (8%)	
**BMI (kg/m^2^), mean ± SD**	22.59 ± 4.15	21.21 ± 3.46	0.309
**BSA (m^2^), mean ± SD**	1.59 ± 0.16	1.52 ± 0.15	0.162
**Patients with prior exposure to neurotoxic chemotherapy, n (%)**	7 (19%)	0 (0%)	0.175
**Cumulative dose of paclitaxel, mg/m^2^, mean ± SD**	993.77 ± 110.32	1039.56 ± 23.95	0.47
**Cumulative dose of carboplatin, mg/m^2^, mean ± SD**	2496.87 ± 725.81	2506.40 ± 2995.61	0.949
**Combination with bevacizumab, n (%)**	3 (8%)	0 (0%)	0.573
**Diabetes mellitus, n (%)**	1 (3%)	1 (8%)	1.0
**EORTC QLQ-CIPN20 scores at pre-chemotherapy, mean ± SD**	19.71 ± 1.23	20.25 ± 1.96	0.389
**EORTC QLQ-CIPN20 scores after two cycles, mean ± SD**	26.03 ± 5.27	29 ± 9.19	0.455
**EORTC QLQ-CIPN20 scores after four cycles, mean ± SD**	28.46 ± 7.81	43.45 ± 10.38	**< 0.001**
**EORTC QLQ-CIPN20 scores after six cycles, mean ± SD**	26 ± 7.73	40.36 ± 7.43	**< 0.001**
**EORTC QLQ-CIPN20 scores after 6 months, mean ± SD**	21.09 ± 3.14	28.2 ± 7.36	**0.009**
**NCI-CTC grade at 6 months after the end of chemotherapy**			**< 0.001**
** Grade 0–1**	31 (86%)	1 (8%)	
** Grade 2**	4 (11%)	8 (67%)	
** Grade 3**	1 (3%)	3 (25%)	

P < 0.05 are shown in bold.

PIPN, paclitaxel-induced peripheral neuropathy; SD, standard deviation; EORTC QLQ-CIPN20, European Organisation for Research and Treatment of Cancer Quality of Life Questionnaire-Chemotherapy-Induced Peripheral Neuropathy 20 module; BMI, body mass index; BSA, body surface area; NCI-CTC, National Cancer Institute-Common Toxicity Criteria.

### Clinical characteristics and NCS results

**Figure 2:**
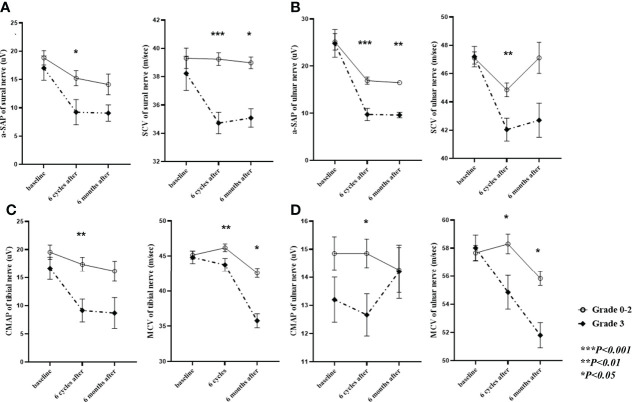
Nerve conduction studies in grade 3 vs. grade 0–2 PIPN patients. Shown is a comparison of a-SAP and SCV in the **(A)** sural and **(B)** ulnar nerves and of CMAP and MCV in the **(C)** tibial and **(D)** ulnar nerves between patients with grade 3 and grade 0–2 paclitaxel-induced neuropathy. The data collected at baseline, during, and 6 months after paclitaxel treatment have been adjusted for age and presented as mean ± standard error. PIPN, paclitaxel-induced peripheral neuropathy; a-SAP, amplitude of the sensory nerve action potential; SCV, sensory nerve conduction velocity; CMAP, compound muscle action potential; MCV, motor nerve conduction velocity.

Patients with grade 3 PIPN were older than those within the 0–2 grade range (p = 0.005) (**Table 1**). No significant differences were observed regarding the type of gynecological cancer, dose of paclitaxel, number of patients receiving bevacizumab co-treatment, incidence of diabetes, and prior exposure to neurotoxic chemotherapy between patients with different grades of PIPN (**Table 1**). Grade 3 PIPN patients had higher total EORTC-QLQ-CIPN20 scores than those within the 0–2 grade range after four and six cycles of treatment, and the difference was still significant 6 months after the end of treatment (**Table 1**). Six months after the end of treatment, 25% of grade 3 PIPN patients retained the same status, and 3% of those with grade 0–2 PIPN had deteriorated to grade 3 PIPN (p < 0.001). Compared to patients within the 0–2 grade range, those with grade 3 PIPN had lower CMAP and MCV of the ulnar and tibial nerves and lower a-SAP and SCV of the ulnar and sural nerves. Most of these differences in the NCS results between grade 3 PIPN and grade 0–2 PIPN patients persisted 6 months after the end of treatment (**Figure 2**).

### Changes in sNfL and sBDNF levels and severity of PIPN

**Figure 3:**
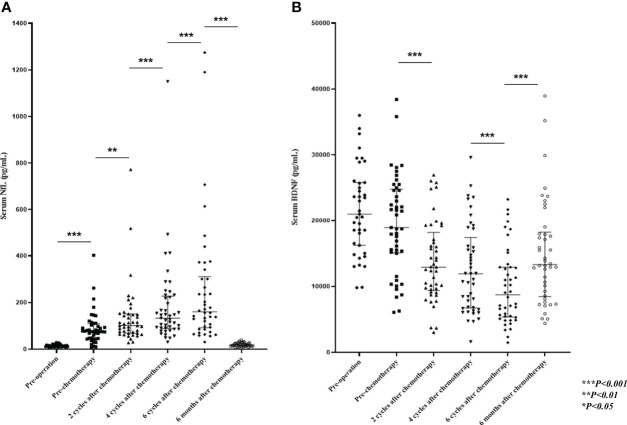
Evaluation of sNfL and sBDNF levels. Shown are the median (IQR), **(A)** sNfL, and **(B)** sBDNF levels during and 6 months after paclitaxel treatment. P-values were calculated using the Wilcoxon signed-rank test. sNfL, serum neurofilament light chain; sBDNF, serum brain-derived neurotrophic factor; IQR, interquartile range.

**Figure 4:**
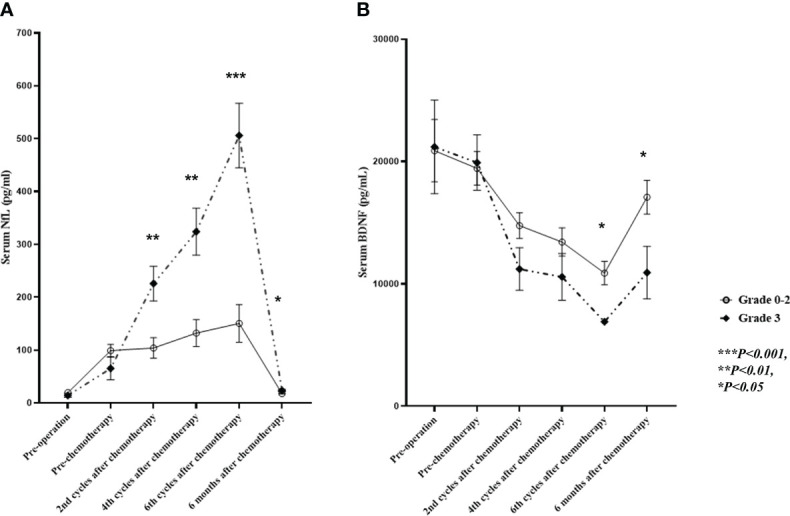
Comparison of sNfL and sBDNF levels in grade 3 vs. grade 0–2 PIPN patients. Shown are the **(A)** sNfL levels, and **(B)** sBDNF levels for patients with grade 3 and grade 0–2 PIPN during and 6 months after paclitaxel treatment. Data are presented as mean ± standard error, and the sNfL comparison has been adjusted for patient age. sNfL, serum neurofilament light chain; sBDNF, serum brain-derived neurotrophic factor; PIPN, paclitaxel-induced peripheral neuropathy.

The pre-chemotherapy sNfL levels (at a median of 20 days after surgery) were higher than the preoperative levels, probably due to nerve injury in the pelvic cavity from radical surgery (**Figure 3A**). Elevated sNfL cutoff levels were calculated as 3 SD higher than the mean value of the HCs for each age group in the 10-year range (40–49; 26ng/mL, 50–59; 28.9 ng/mL, and 60–69; 36.7ng/mL). The preoperative sNfL levels in 47 (98%) of 48 patients were within the normal range of HCs. The preoperative levels of sNfL, but not sBDNF, increased with age (r = 0.568, p = 0.001). The mean sNfL levels began to increase after two cycles of treatment relative to the pre-chemotherapy levels (p = 0.009) and continued to increase through the remaining course of treatment. However, the levels returned to near-baseline 6 months after the end of treatment (**Figure 3A**); 47 (98%) patients showed sNfL level within the normal range 6 months after the end of treatment. Grade 3 PIPN patients had higher sNfL levels after two, four, and six cycles of treatment compared to those within the 0–2 grade range (p = 0.004, p = 0.001, and p < 0.001, respectively) (**Figure 4A**; **Supplementary Table 1**). After four and six cycles, the ROC curve analysis with a cut-off sNfL level of 196.5pg/mL and 250 pg/mL showed 75% and 90% sensitivity and 84% and 89% specificity for grade 3 PIPN, respectively (**Supplementary Figure 1**). Furthermore, after two cycles, using a cut-off sNfL level of 124 pg/mL, the sensitivity and specificity for predicting grade 3 PIPN at the end of treatment were 80% and 79%, respectively. After six cycles, the sNfL levels positively correlated with the EORTC QLQ-CIPN20 sensory (r = 0.389, p = 0.011) and motor (r = 0.404, p = 0.008) scores, and negatively correlated with the SCV of the sural nerve (r = -0.361, p=0.02), the SCV of the ulnar nerve (r =-0.333, p = 0.033), the CMAP of the tibial nerve (r = -0.323, p = 0.04), the MCV of the tibial nerve (r = -0.414, p = 0.007), and the CMAP of the ulnar nerve (r = -0.402, p = 0.009). The sBDNF levels started decreasing after two treatment cycles and continued this trend over the entire course of the treatment (**Figure 3B**). During the treatment course, sBDNF levels were comparable between patients with grade 3 PIPN and those within the 0–2 grade range (**Figure 4B**). However, after six cycles and 6 months post-chemotherapy, grade 3 PIPN patients had lower sBDNF levels than the rest of the patients (p = 0.037 and 0.02, respectively) (**Figure 4B**; **Supplementary Table 1**). Platelets are the major source of peripheral BDNF, and, therefore, sBDNF levels are positively associated with platelet counts (16). Platelet counts showed a decrease in counts throughout the treatment, which remained lower than baseline at 6 months after treatment. Unlike sBDNF levels, platelet counts were comparable between patients with grade 0–2 and grade 3 PIPN throughout the treatment and 6 months after the end of treatment (**Supplementary Figure 2**). Thus, the significant differences in sBDNF levels after six cycles and 6 months post-chemotherapy between patients with grade 3 PIPN and grade 0–2 PIPN was not due to difference in platelet counts.

## Discussion

We have observed a significant increase in sNfL concentration over the course of paclitaxel treatment and its correlation with disease severity assessed by electrophysiological studies and clinical tools, suggesting that sNfL levels could be indicative of PIPN severity. The return of sNfL levels to near-normal 6 months after the end of treatment suggests that no further axonal injury occurred after the cessation of chemotherapy. Thus, sNfL levels during paclitaxel treatment reflect the ongoing neuroaxonal injury. These findings are in line with those of our previous study, in which we proposed sNfL as a biomarker to monitor neuroaxonal injury in OIPN (7).

The progressive increase in the severity of clinical symptoms and sNfL levels over the course of paclitaxel treatment confirms the typical dose-dependent nature of PIPN. Nevertheless, no significant difference in the cumulative paclitaxel dose between patients with grade 0–2 and grade-3 PIPN suggests the involvement of other patient-specific factors. Older patients appear to be at a greater risk of severe PIPN. While some previous studies have reported an increased risk of developing CIPN in older patients (17, 18), others found no such association (19, 20). Consistent with previous studies, a combination of bevacizumab with paclitaxel plus carboplatin did not increase the incidence of severe PIPN in our cohort (21, 22). In contrast to a recent large multi-center study (17), we found no significant association between body mass index and PIPN severity. Previous studies have reported prior exposure to neurotoxic agents as a risk factor for PIPN (23), but none of our seven patients with prior exposure developed grade 3 PIPN. These patients had no or mild neuropathic symptoms during the previous chemotherapy round, indicating their tolerance to neurotoxicity, which may explain why they were not at an increased risk of developing severe neuropathy despite additional paclitaxel-based chemotherapy.

Although several demographic and clinical factors are known risk factors for PIPN, predicting its occurrence and severity remains a challenge in individual patients. Notably, after two cycles, there were no significant differences in the patient-reported symptoms (EORT QLQ-CIPN20 scores) in those with grade 3 vs. grade 0–2 PIPN (at the end of therapy). However, compared to the grade 0–2 patients, the levels of sNfL were significantly higher in the grade 3 PIPN group (mean 250.2 vs. 94.7 pg/mL, p = 0.004), with a much steeper increase throughout the treatment. Meanwhile, in our previous study on OIPN, the sNfL levels measured during oxaliplatin treatment did not predict the risk of severe OIPN at the end of treatment (7). In PIPN, pronounced neuronal injury appears to develop early in those patients who are more susceptible to paclitaxel neurotoxicity. Genetic variants associated with increased sensitivity to taxane-induced neuropathy support this hypothesis (24). Therefore, high sNfL levels (> 124 pg/mL) after two cycles of paclitaxel may help predict patients at risk of developing severe PIPN after completion of chemotherapy.

At the end of the treatment, patients with grade 3 PIPN had higher sNfL levels (mean: 506 pg/mL) than patients with grade 3 OIPN in our previous study (mean: 373.4 pg/mL) (7). PIPN primarily affects large myelinated Aβ-fibers, which is reflected by symptoms such as the impaired sensation of vibration or touch (13), while oxaliplatin mainly affects the small myelinated Aδ fibers related to the sensation of cold and pain (25). Furthermore, while oxaliplatin treatment had no significant effect on the CMAP of the motor nerves in the NCS (7), a reduction was observed after paclitaxel treatment. Therefore, it appears that axonal injury in large-caliber myelinated fibers results in higher sNfL levels in PIPN patients than in OIPN patients due to the abundance of sNfL in large-caliber fibers. These results are consistent with those of previous studies in animal models showing higher sNfL concentrations and a significant reduction in a-SAP in paclitaxel-treated than in cisplatin-treated animals (26).

Because NCS predominantly identifies already acquired axonal damage, lower a-SAP and CMAP even at 6 months after chemotherapy compared to baseline reflect the non-recovery of damaged axons during chemotherapy. Consistently, in our previous study on long-term outcome of OIPN, no significant recovery of the a-SAP of sensory nerves was found 6 months after the end of treatment (6). Meanwhile, sNfL has a half-life of several weeks to one or two months (27, 28), which means that a normal range of sNfL at 6 months after end of chemotherapy in most patients suggests absence of further ongoing neuroaxonal injury. Longitudinal studies on other neurological diseases reported that sNfL levels and their change over time correlated with the clinical course of the diseases. Elevated levels of sNfL return to normal several months after stroke or traumatic brain injury, but remain relatively stable throughout the course of neurodegenerative diseases (29). The temporal dynamics of sNfL level during and after chemotherapy in the current study suggest that serial monitoring of sNfL levels could be a useful biomarker of ongoing neuroaxonal injury during chemotherapy.

Although neurotoxicity could result from the overall drug exposure, the mechanisms underlying PIPN are related, at least in part, to the ability to repair nerve damage. BDNF augments neurogenesis and improves synaptic plasticity (10), implying its role in the pathogenesis of PIPN. Low sBDNF levels have been associated with the development of diabetic polyneuropathy and severe CIPN after vincristine or bortezomib-based therapy (11, 30). In the present study, the mean sBDNF levels decreased with paclitaxel treatment. Given that platelets are the most critical peripheral reservoir of BDNF (31), it is likely that the decrease in sBDNF levels may be an epiphenomenon resulting from a paclitaxel-induced decrease in platelet counts. However, after six cycles of chemotherapy and 6 months after treatment, patients with grade 3 PIPN (at the end of treatment) showed lower sBDNF levels than grade 0–2 PIPN patients, although there was no significant difference in their platelet counts. Thus, a significant decrease in sBDNF levels after 6 cycles of chemotherapy may contribute to development of severe PIPN. In addition, based on the delayed recovery of CIPN symptoms after discontinuing chemotherapy in grade 3 CIPN patients, the persistently low sBDNF levels 6 months after chemotherapy might negatively affect CIPN recovery, although these findings need further validation.

This study has some limitations. First is the limited number of patients and that they all belonged to a single race category. Second, while paclitaxel is used to treat breast, lung, and pancreatic cancer, this study focuses on gynecological cancer. Therefore, extrapolation of our findings to other types of cancer warrants confirmation. Third, sNfL levels can increase in response to any neuroaxonal damage, not specifically PIPN, limiting its use for PIPN diagnosis. Finally, we could not determine how much carboplatin contributed to the development of neuropathy and the increase in sNfL, although carboplatin-induced peripheral neuropathy is rare at conventional doses. Despite these limitations, we believe that sNfL levels during paclitaxel-based chemotherapy using the highly sensitive SIMOA technique reflect ongoing neuroaxonal injury and serve as reliable biomarkers for the severity of PIPN. Furthermore, sNfL levels during early treatment with paclitaxel might be useful prognostic indicators for severe PIPN progression. Hence, this objective and quantitative biomarker can help monitor the response to treatment in future clinical trials of preventive or therapeutic agents for CIPN and identify patients at high risk of developing severe PIPN. Lower sBDNF levels after paclitaxel discontinuation may be associated with a risk of poor recovery from PIPN. Prospective studies including larger patient populations are needed to verify our observations.

## Data availability statement

The original contributions presented in the study are included in the article/**Supplementary Material**. Further inquiries can be directed to the corresponding author.

## Ethics statement

The studies involving human participants were reviewed and approved by the institutional review board of the National Cancer Center. The patients/participants provided their written informed consent to participate in this study.

## Author contributions

S-HK and MCL had full access to all the data in the study and take responsibility for the integrity of the data and the accuracy of the data analysis. Study concept and design, S-HK and MCL. Acquisition, analysis or interpretation of data, S-HK, KK, J-WH, JK, S-SS, S-YP, HK, and MCL. Drafting of manuscript, S-HK. Critical revision of manuscript for important intellectual content, S-HK, KK, J-WH, JK, S-SS, S-YP, HK, and MCL. Statistical analysis and interpretation, S-HK, HK, and MCL. Obtained funding, S-HK. All authors contributed to the article and approved the submitted version.

## Funding

This work was funded by the National Research Foundation of Korea (Grant No. 2020R1F1A1075345).

## Acknowledgments

We thank Ji-Hee kim, BSc and Min Ki Woo, BSc for assistance with electrophysiological procedures. Preoperative blood samples from 32 study participants with gynecological cancer were provided by the National Cancer Center (NCC) Biobank.

## Conflict of interest

S-HK received a grant from the National Research Foundation of Korea and consultancy/speaker fees from Eisai, Merck Serono, Roche, and Sanofi Genzyme. HK received a grant from the National Research Foundation of Korea and research support from Aprilbio and Eisai, received consultancy/speaker fees from Alexion, Aprilbio, Biogen, Celltrion, Daewoong, Eisai, GC Pharma, HanAll BioPharma, MDimune, Merck Serono, Novartis, Roche, Sanofi Genzyme, Teva-Handok, UCB, and Viela Bio, is a co-editor for the Multiple Sclerosis Journal and an associated editor for the Journal of Clinical Neurology. S-YP reported having a consulting or advisory role for Boryung and Takeda. MCL reported having a consulting or advisory role for AstraZeneca, Boryung, CKD Pharm, Genexine, Hospicare, GI Innovation and Takeda and received research funding from AbbVie, Amgen, Astellas, AstraZeneca, BeiGene, Cellid, CKD Pharm, Clovis, Eisai, Genexine, GSK, Incyte, Merck, MSD, OncoQuest, Pfizer, and Roche outside the submitted work.

The remaining authors declare that the research was conducted in the absence of any commercial or financial relationships that could be construed as a potential conflict of interest.

## Publisher’s note

All claims expressed in this article are solely those of the authors and do not necessarily represent those of their affiliated organizations, or those of the publisher, the editors and the reviewers. Any product that may be evaluated in this article, or claim that may be made by its manufacturer, is not guaranteed or endorsed by the publisher.

## Abbreviations

a-SAP, sensory nerve action potential; BDNF, brain-derived neurotrophic factor; CIPN, chemotherapy-induced peripheral neuropathy; CMAP, compound muscle action potential; EORTC QLQ-CIPN20, European Organisation for Research and Treatment of Cancer Quality of Life-Chemotherapy-Induced Peripheral Neuropathy 20 module; HC, healthy control; IQR, interquartile range; IV, intravenously; MCV, motor nerve conduction velocity; NCI-CTC, National Cancer Institute-Common Toxicity Criteria; NCS, nerve conduction study; NfL, neurofilament light chain; OIPN, oxaliplatin-induced peripheral neuropathy; PIPN, paclitaxel-induced peripheral neuropathy; ROC, Receiver operating characteristics; sBDNF, serum brain-derived neurotrophic factor; SCV, sensory nerve conduction velocity; SD, standard deviation; SIMOA, single molecule array platform; sNfL, serum neurofilament light chain.
