# Corrigendum: Blood neurofilament light chain as a biomarker for monitoring and predicting paclitaxel-induced peripheral neuropathy in patients with gynecological cancers

**DOI:** 10.3389/fonc.2023.1279558

**Published:** 2023-09-07

**Authors:** Su-Hyun Kim, Ki Hoon Kim, Jae-Won Hyun, Ji Hyun Kim, Sang-Soo Seo, Ho Jin Kim, Sang-Yoon Park, Myong Cheol Lim

**Affiliations:** ^1^ Department of Neurology, Research Institute and Hospital of National Cancer Center, Goyang, Republic of Korea; ^2^ Center for Gynecologic Cancer, National Cancer Center, Goyang, Republic of Korea; ^3^ Center for Clinical Trial, Hospital, National Cancer Center, Goyang, Republic of Korea; ^4^ Department of Cancer Control and Population Health, National Cancer Center Graduate School of Cancer Science and Policy, Goyang, Republic of Korea; ^5^ Rare and Pediatric Cancer Branch and Immuno-oncology Branch, Division of Rare and Refractory Cancer, Research Institute, National Cancer Center, Goyang, Republic of Korea; ^6^ Department of Cancer Control and Policy, National Cancer Center, Goyang, Republic of Korea

**Keywords:** paclitaxel, neuropathy, neurofilament light (NfL), gynecological cancer, chemotherapy induced peripheral neuropathy

In the published article, acknowledgement was mistakenly not included in the publication. The missing material appears below:

“Preoperative blood samples from 32 study participants with gynecological cancer were provided by the National Cancer Center (NCC) Biobank.”

The authors apologize for this error and state that this does not change the scientific conclusions of the article in any way. The original article has been updated.

