# Supplementary material for: Blood neurofilament light chain as a biomarker for monitoring and predicting paclitaxel-induced peripheral neuropathy in patients with gynecological cancers
Source: Front Oncol. 2022 Aug 17;12:942960. doi: 10.3389/fonc.2022.942960 (PMC9428708; doi:10.3389/fonc.2022.942960)

Supplementary figure 1. ROC curve analysis of the sNfL levels for predicting grade 3 PIPN.

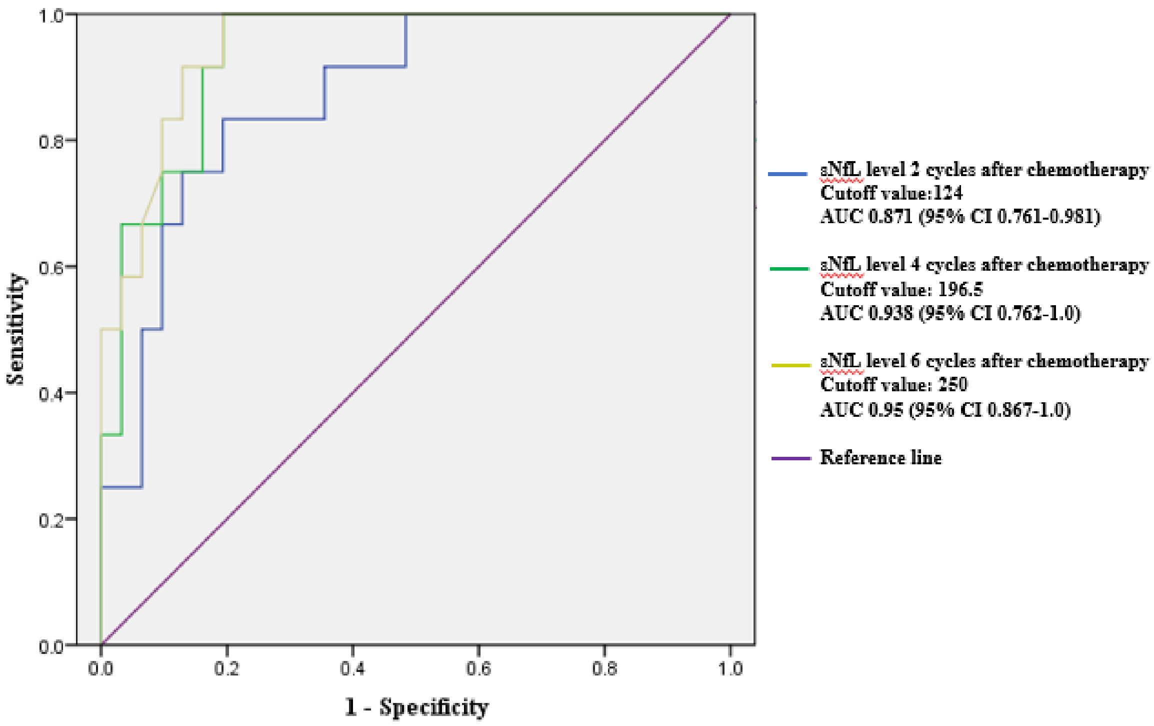

PIPN: paclitaxel-induced peripheral neuropathy, sNfL: serum neurofilament light chain, AUC: area under the receiver operating characteristic curve

Supplementary figure 2. Platelet counts

Serial platelet counts during and six months after paclitaxel treatment in (A) the total study population and (B) grade-3 vs. grade 0–2 PIPN patients. Data are presented as (A) median (IQR) and (B) mean and standard error values.

PIPN: paclitaxel-induced peripheral neuropathy, PLT: platelets.

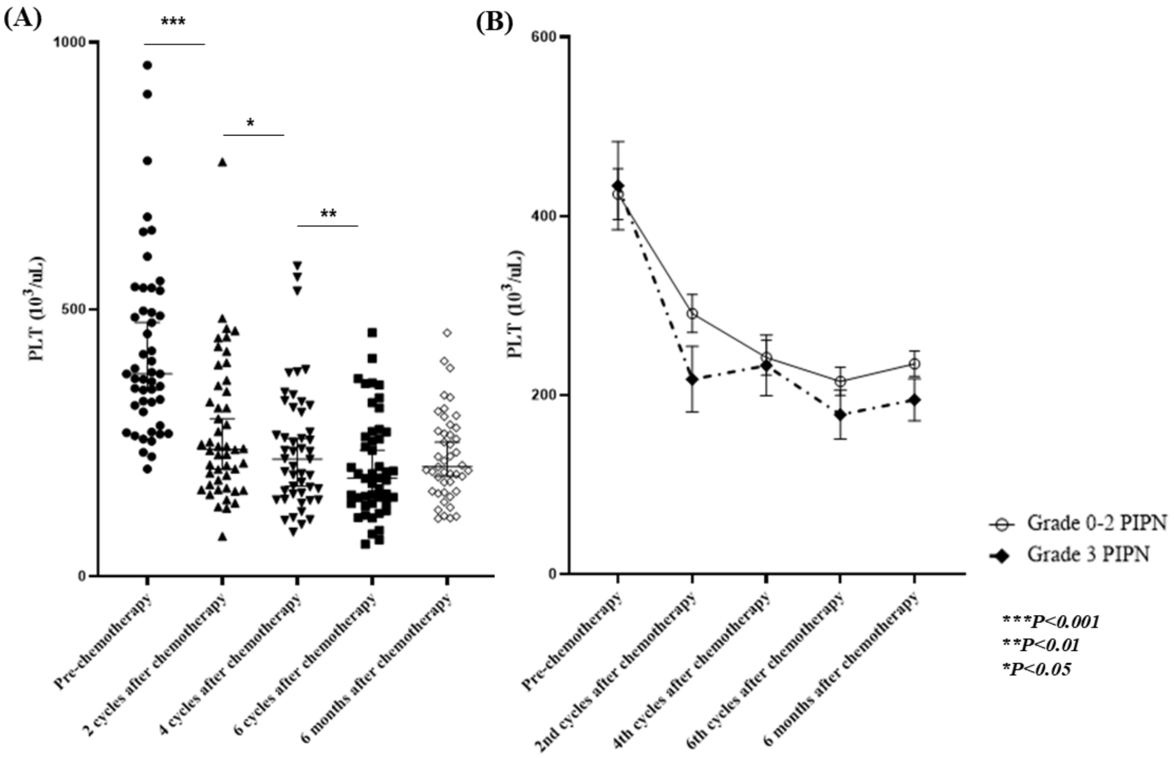

Supplement: Supplementary file 1 [file DataSheet_1.pdf]
