# Supplementary material for: Blood neurofilament light chain as a biomarker for monitoring and predicting paclitaxel-induced peripheral neuropathy in patients with gynecological cancers
Source: Front Oncol. 2022 Aug 17;12:942960. doi: 10.3389/fonc.2022.942960 (PMC9428708; doi:10.3389/fonc.2022.942960)
Supplement: Supplementary file 2 [file Table_1.pdf]

Supplementary Table 1 Comparison of sNfL and sBDNF levels in grade 3 vs. grade 0-2 PIPN patients.

	Serum NfL level (pg/mL), mean (SE)			Serum BDNF level (pg/mL) (mean SE)		
	Patients with grade 0-2 PIPN	Patients with grade 3 PIPN	p-value	Patients with grade 0-2 PIPN	Patients with grade 3 PIPN	p-value
Pre-op	18.2 (2.1)	20.3 (3.5)	0.627	21268 (2254)	20425 (3187)	0.833
Pre-chemotherapy	98.9 (12)	65.3 (21.8)	0.2	19437 (1367)	19912 (2267)	0.859
2nd cyles after chemotherapy	103.9 (19.4)	225.8 (32.9)	**0.004**	14757 (1046)	11211 (1735)	0.087
4th cycles after chemotherapy	132.2 (22.6)	324.1 (44.3)	**0.001**	13418 (1150)	10564 (1908)	0.207
6th cycles after chemotherapy	150.4 (35.8)	506. (60.9)	**<0.001**	10877 (950)	6906(1576)	**0.037**
6 months after chemotherapy	17.6 (1.2)	23.5 (2)	**0.022**	17086 (1379)	10920 (2144)	**0.02**

$P < 0.05$ are shown in bold.

sNfL comparison has been adjusted for patient age.

sNfL: serum neurofilament light chain, sBDNF: serum brain-derived neurotrophic factor, PIPN: paclitaxel-induced peripheral neuropathy
